# Effect of Temperature on Material Properties of Carbon Fiber Reinforced Polymer (CFRP) Tendons: Experiments and Model Assessment

**DOI:** 10.3390/ma12071025

**Published:** 2019-03-28

**Authors:** Fei Zhou, Jiwen Zhang, Shoutan Song, Dong Yang, Chao Wang

**Affiliations:** 1 School of Civil Engineering, Southeast University, Nanjing 210096, China; zhoufei0912@seu.edu.cn (F.Z.); songshoutan@seu.edu.cn (S.S.); dongyang@seu.edu.cn (D.Y.); chao.wang.seu@gmail.com (C.W.); 2 Key Laboratory of Concrete and Prestressed Concrete Structures of the Ministry of Education, Southeast University, Nanjing 210096, China

**Keywords:** carbon fiber reinforced polymer, material properties, experimental tests, elevated temperatures, modeling

## Abstract

Material properties at elevated temperatures are important factors in the fire safety design and numerical analysis of concrete members strengthened with fiber reinforced polymer (FRP) composites. Most of the previous research mainly focused on tensile strength and elastic modulus in conventional steady state temperature tests. However, the transient state test method is more realistic for strengthening concrete structures. At the same time, the coefficient of thermal expansion of FRP composites is also one of the important factors affecting concrete members at elevated temperatures. This paper presents a detailed experimental investigation on the longitudinal thermal expansion deformation, and the mechanical properties of carbon FRP (CFRP) tendons with 8 mm diameter in the steady state and transient state. The results indicate that longitudinal deformation of CFRP tendons is negative at high temperature; in addition, the transient state test results of CFRP tendons are slightly higher than the steady state test results. The final part of this paper assesses the accuracy of different empirical models. Furthermore, a new equation calculating the properties of CFRP composites at elevated temperatures is presented with the numerical fitting technique, which is in good agreement with the experimental results.

## 1. Introduction

In recent years, the application of fiber reinforced polymer (FRP) composites has increased significantly in civil engineering structures around the world. It is mainly attributed to the fact that FRP composites have many advantages compared with steel, such as high strength-to-weight ratio, corrosion resistance, fatigue resistance, and non-magnetic properties. Although numerous investigations have shown that FRP composites are effective materials for concrete members [1,2], many challenges still exist in the increasing application of FRP composites. One of these challenges is to fully understand the material properties of FRP composites at elevated temperatures. Due to the fact that FRP composites are typically produced from the thermosetting polymer matrix, their mechanical properties deteriorate severely when exposed to fire. Even though FRP composites embedded in the concrete do not burn due to lack of oxygen, the resin will soften and decompose when the temperature exceeds the glass transition temperature (*T_g_*) and the decomposition temperature (*T_d_*). The softening and decomposition of the resin not only reduces the strength of the resin itself, but also weakens the bonding effect of fibers, resulting in the rapid reduction of the strength of FRP composites.

The research conducted by Yu Bai [3,4], has indicated that the mechanical properties of FRP composites are affected significantly by their thermal properties when subjected to elevated and high temperatures. Furthermore, the thermo–mechanical behavior of FRP composites depends mainly on the states (glassy, leathery, rubbery, and decomposed) of the polymer resin at high temperature. In the experimental study of glass FRP (GFRP) composites, it was also confirmed that the elastic modulus and strength decreased significantly at raised temperatures, which reached the range of glass transition and decomposition temperatures [5]. Similarly, Yu Baolin [6] tested two types of near surface mounted (NSM) carbon FRP (CFRP) composites. It was found that the strength of both CFRP strip and rod dropped dramatically when the temperature reached and exceeded *T_g_* (around 80 °C) and *T_d_* (around 300 °C). Since the low *T_g_* of resin is the greater shortcoming of FRP composites at elevated temperatures, an improved resin with a higher *T_g_* was tested by Zhu [7]. After improving the *T_g_* of basalt FRP (BFRP) composites, the concrete beams strengthened with near surface mounted (NSM) BFRP bars exhibited better fire resistance. Therefore, the comprehensive study of the high temperature characteristics of FRP composites can greatly improve the fire resistance of concrete members strengthened with FRP composites.

In order to further understand the inherent properties of FRP composites at high temperature, Saafi M [8] suggested constitutive models for the mechanical properties of aramid FRP (AFRP) rebars, CFRP rebars, and glass FRP (GFRP) rebars linearly decreasing with temperature according to the experimental results of Blontrok [9]. However, a large amount of experimental research has shown that the tensile strength and elasticity modulus of FRP composites do not deteriorate linearly with temperature [10,11,12]. Therefore, Bisby [13] proposed a hyperbolic tangent function model based on the experimental data of relevant literature. The fitting coefficients were obtained by least square regression analysis. In order to fully reveal the influence of thermo–physical responses on thermo–mechanical properties, Gibson [14] successfully applied the glass transition temperature (*T_g_*) and remaining resin content (RRC) criteria to the hyperbolic tangent function model. Subsequently, the Gibson’s model was modified by Yu [6] and Chowdhury [11], respectively. It is considered that *T_g_* in Gibson’s model can be the critical temperature or the temperature around which the curve is nearly symmetrical rather than the glass transition temperature. Yu [6] carried out experimental testing of CFRP bars and CFRP slabs, and fitted the obtained results with the hyperbolic tangent function model. Nevertheless, with the improvement of the measurement methods, the curve of the mechanical properties was more and more similar to that of thermo–physical state of FRP composites at high temperatures [15,16]. Yu Bai [3] believed at any specific temperature, a composite material could be considered to be a mixture of materials in different states, with different mechanical properties. Wang [17] also confirmed Bai’s theory by testing the mechanical properties of GFRP bars at high temperature. The exponential function model was proposed for the deterioration of ultimate tensile strength. However, the elastic modulus of GFRP bars decreased with temperature, which did not meet with Wang’s model. From the previous research it can be seen that the fitting model based on the material properties of FRP composites at high temperature still needs further discussion. A unified expression with better fitting accuracy can greatly facilitate the numerical analysis of strengthened structures.

In addition, most of the previous research mainly focused on tensile strength and elastic modulus in conventional steady state temperature tests. However, the transient state test method is more realistic for application. At the same time, the coefficient of thermal expansion (CTE) for FRP composites is also one of the important factors affecting reinforced concrete members at high temperatures. Therefore, this paper presents a detailed experimental investigation on the longitudinal thermal expansion deformation, and the mechanical properties of CFRP tendons with 8 mm diameter in the steady state and transient state. Subsequently, a new constitutive model of CFRP tendons at high temperature is proposed based on the analysis of thermophysical properties.

## 2. Experimental Program

### 2.1. Material Characteristics and Specimen Details

In the current research, CFRP tendons with 8 mm diameter and smooth surface from Jiangsu Hengshen Co. Ltd. (Zhenjiang, China) were tested. They were made of T700 continuous carbon fibers impregnated in thermosetting epoxy resin with a fiber content of 65%. The average tensile strength and modulus of CFRP tendons were 2070 MPa and 156 GPa, respectively, at room temperatures. According to E1356-08 [18], The *T_g_* value is usually the midpoint temperature of resin glass transition. The *T_d_* value can be considered as the temperature point where the weight loss of CFRP composites reaches the peak (the peak point of DTG curve) [19]. Based on the previous experimental study, the *T_g_* and *T_d_* of epoxy resins from the same batches were 126 °C and 405 °C, respectively [20]. Other properties of CFRP tendons used for testing are listed in Table 1.

**Figure 1:**
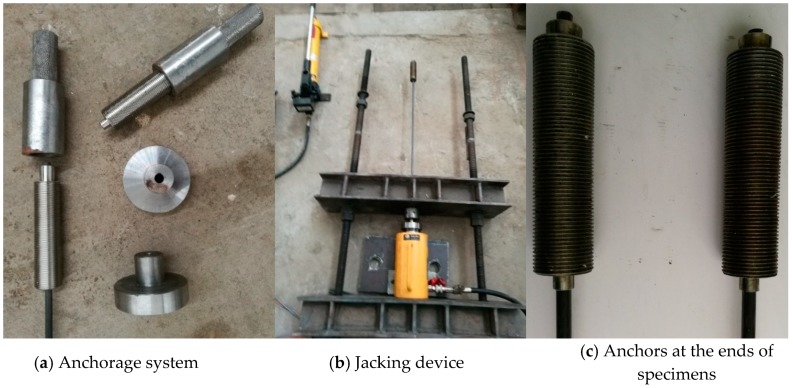
Anchorage system for CFRP tendons.

In the tensile test, special anchorage was needed at both ends of the specimen due to the smooth surface of the tendons. So far, the clip anchorage is the most reliable anchorage for CFRP tendons, which can effectively prevent the slippage between the tendon and clip by pre-pressing (Figure 1). Details of the anchorage system for CFRP specimen were presented in Zhou [20].

### 2.2. Testing Setups

**Figure 2:**
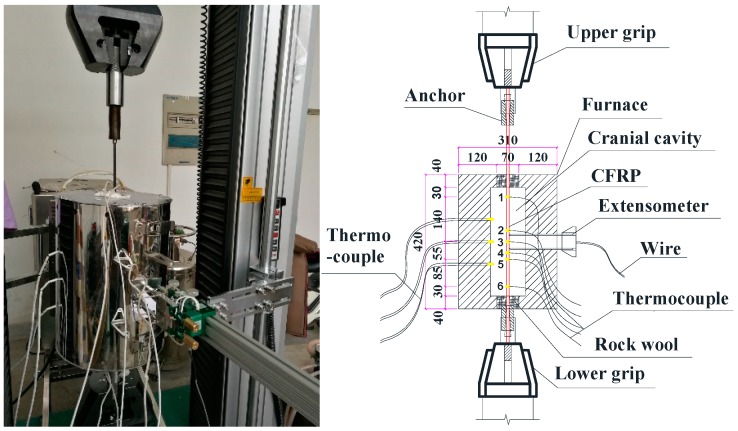
Test setup for CFRP tensile tests at elevated temperatures.

The tests were performed at the Testing Laboratory of Civil Engineering at Southeast University. A UTM5305 Material Test System with a loading capacity of 300 kN was used in this study, as shown in Figure 2. The tendons used in the tests were all 800 mm long, of which 340 mm was exposed to heat inside the furnace. The elongation of CFRP tendons was measured by a high temperature extensometer with a gauge length of 50 mm. In order to obtain more accurate results, the temperature distribution on the surface of the tendons was measured by six thermocouples fixed on the surface of the tendon.

### 2.3. Testing Procedure

The tests of the material properties of CFRP tendons at elevated temperatures were divided into three series, namely thermal expansion test, steady state test, and transient state test. A total of 42 specimens were performed in this study, as shown in Table 2.

(1) Thermal expansion tests

**Figure 3:**
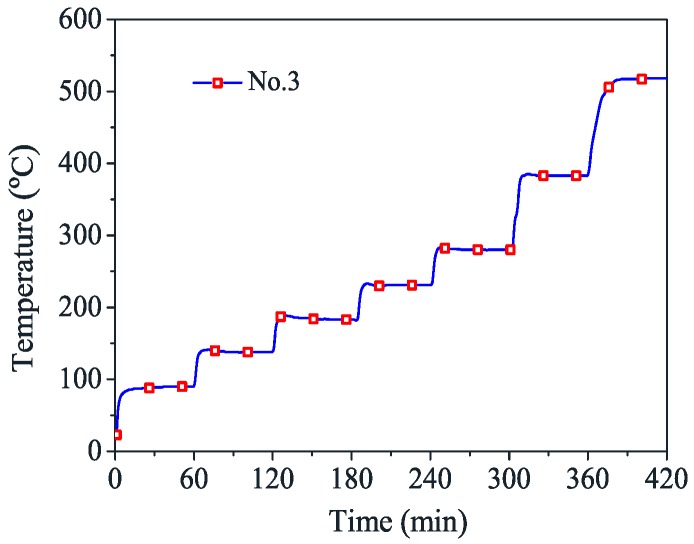
Time–temperature curve for no. 3 point.

**Figure 4:**
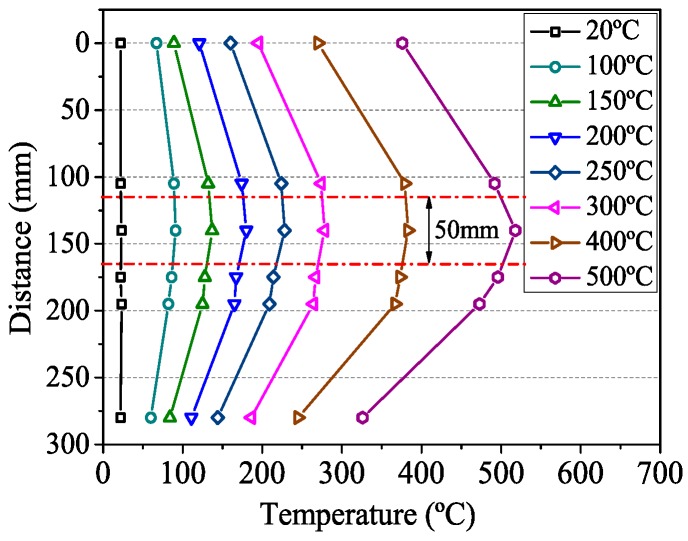
Constant temperature distribution on the surface of CFRP tendons.

In the thermal expansion test, one end of the specimen was gripped by the upper grip, and the other end was relaxed. In order to make the outer and inner temperatures of the tendons to be consistent, the furnace temperature (target temperature) was held constant for about half an hour after it was raised to a target temperature (Figure 3) [10]. The surface temperature of CFRP tendons detected by six thermocouples is presented in Figure 4. It shows that the temperature distribution at the specimen surface was not uniform. The temperature difference within the 50 mm range of the extensometer was small, and the maximum temperature value appeared near the no. 3 point. Thus, the temperature at no. 3 point was chosen as the failure temperature. Finally, when the temperature was stable, the longitudinal deformations of CFRP tendons were recorded by the extensometer.

(2) Steady state tests

In order to avoid the influence of free expansion, the installation process at the beginning of the steady state test was similar to that of expansion test. The specimens were exposed to the target temperature for additional 30 min as soak time. The temperature distribution on the surface of the tendons was similar to that shown in Figure 4. Then, the lower grip was tightened. According to the ACI 440.3R [21], the specimens were loaded with displacement control at a rate of 3 mm/min (585 MPa/min) until failure.

(3) Transient state tests

In the transient test, the specimens were first applied to a target load at a rate of 3 mm/min (585 MPa/min). After load stabilization for 1 min, the temperature was raised at a heating rate of 20 °C/min while maintaining the pre-set load until failure occurred. According to GB 50608-2010 [22], the allowable service stress of CFRP bars is limited to not more than 71% of the ultimate tensile strength. Therefore, 29–67% of the ultimate tensile strength at room temperature was chosen as the stress levels for the loaded specimens in this study.

## 3. Discussion of the Test Results

### 3.1. Thermal Expansion Tests

**Figure 5:**
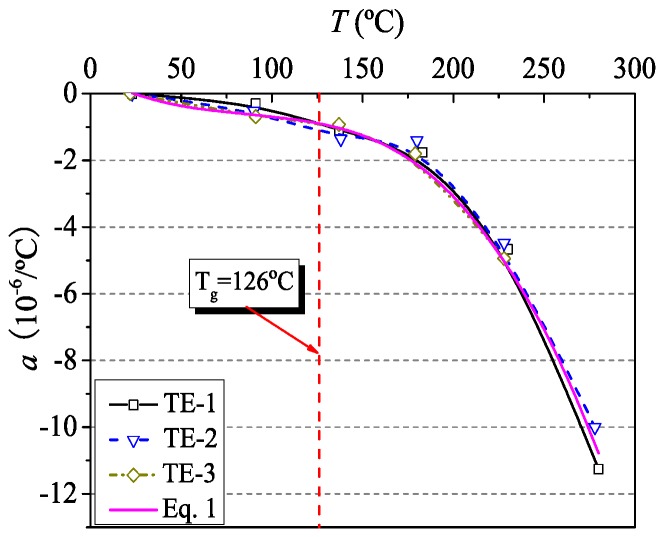
Expansion coefficient–temperature curves.

According to the ACI 440.1R [23], the coefficient of thermal expansion (CTE) of CFRP tendons is between −9.0 × 10^−6^/°C and 0.0 × 10^−6^/°C in the longitudinal direction. However, the variation of CTE with temperatures is not clear. The experimental results show that the longitudinal deformation of CFRP tendons decreased with the increase of temperature (Figure 5), which verifies the fact that the FRP composites shortened along the fiber direction at elevated temperatures [24]. This is mainly attributed to fact that carbon fibers shrink at elevated temperatures in the longitudinal direction [25]. With the increase of temperature, the shrinkage of carbon fibers dominated the longitudinal deformation of CFRP tendons due to the softening of the resin. When the temperature was low, the CTE decreased slowly. As the temperature rose, the resin of FRP composites began to soften, and the CTE of CFRP tendons decreased to a larger negative value. Especially, when the temperature exceeded 200 °C, the CTE decreased rapidly. Finally, the longitudinal deformation of CFRP tendons was unstable after 300 °C due to the decomposition of the resin, resulting in the failure of obtaining its thermal expansion properties.

Based on the experimental data, the longitudinal CTE (10^−6^/°C) of CFRP tendons is proposed in the form of polynomial function (Equation (1)). The obtained curve is in good agreement with the experimental results as shown in Figure 5.

(1) $$\alpha =-1.5\times 10^{-6}{\left(T-23\right)}^{3}+3.17\times 10^{-4}{\left(T-23\right)}^{2}-2.44\times 10^{-2}(T-23)+0.015$$

### 3.2. Steady State Tests

**Figure 6:**
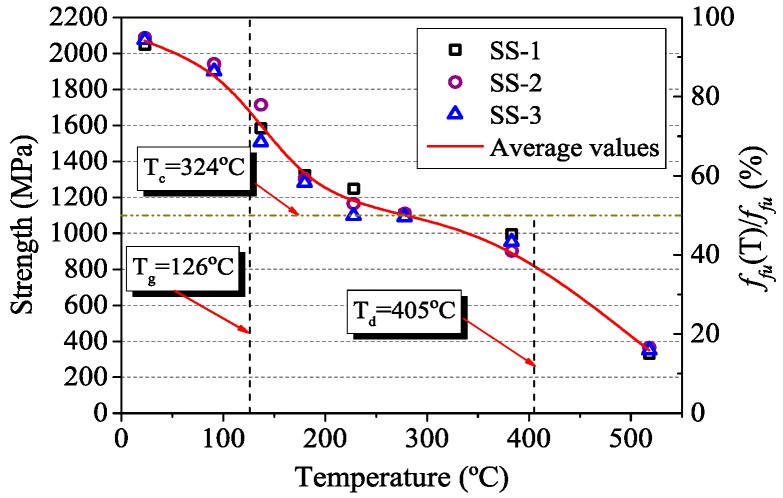
Tensile strength degradation in steady state tests.

In the steady state tests, the experimental results of CFRP tendons at various temperatures are listed in Table 3. It can be seen that maximum temperature on the surface (no. 3 point) of CFRP tendons was less than the furnace temperature (target temperature). When the temperature in the furnace exceeded 500 °C, the surface temperature of CFRP tendons was higher than the furnace temperature because of the combustion phenomenon of CFRP composites. The ultimate tensile strength of CFRP tendons at room temperature was 2070 MPa, but the strength decreased with the increase of temperature, as shown in Figure 6. When the temperature reached and exceeds *T_g_* and *T_d_*, the tensile strength of CFRP tendons decreased dramatically. The main reason is that the softening and decomposition of the resin not only reduced the strength of the resin itself, but also weakened the bonding effect of fibers, resulting in the rapid reduction of the strength of FRP composites. This phenomenon further verifies the effect of temperatures on the mechanical properties of FRP composites due to different state of the resin at elevated temperatures. In order to determine the fire resistance of concrete members strengthened with FRP composites, the temperature at which the composites lose 50% of its tensile strength was used as the critical temperature of FRP composites by Wang [26]. Therefore, the *T_c_* of CFRP tendons was determined to be 324 °C by the linear interpolation method according to Table 3.

**Figure 7:**
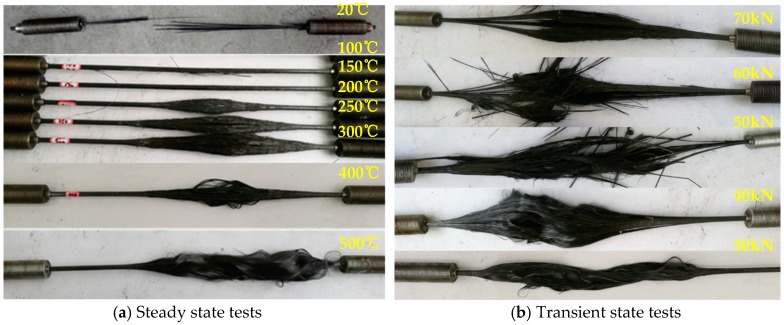
Failure modes of CFRP tendons at elevated temperatures.

Figure 7a shows the failure mode of CFRP tendons in the steady state test. Due to the limitation of the location of the furnace, the heating area was not in the middle of the specimen. However, all specimens failed within the length of the specimen, and the clip anchorage provided effective anchoring effect. In Figure 7a, it is obvious that the temperature distribution of CFRP tendons was not uniform, and the deterioration of resin was more serious near no. 3 point. As the temperature increased, the bonding effect of the resin decreased gradually. This resulted in a gradual separation of the carbon fibers and the resin. At 200–300 °C, there were obvious longitudinal cracks on the surface of the tendons, and the CFRP tendons were divided into several bunches of fibers. When the temperature exceeded 500 °C, only carbon fibers in the CFRP tendons were retained due to decomposition of the resin. At this time, the majority of the mechanical properties of CFRP tendons was lost.

### 3.3. Transient State Tests

**Figure 8:**
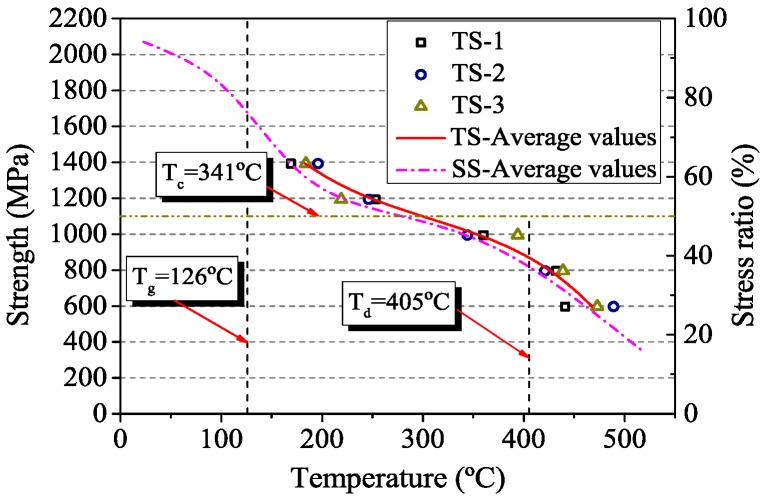
Variation of failure temperatures with target loads in transient state tests.

Table 4 presents failure temperature of the loaded specimens in transient state tests. It should be noted that the failure temperature gradually decreased with the increase of the load level. Corresponding to the steady state test results, the critical temperature of CFRP tendons was 341 °C in the transient test. In theory, the presence of initial stress may intensify and accelerate degradation of CFRP tendons at elevated temperature. However, as shown in Figure 8, most of the data values in transient state tests were slightly higher than those in steady state tests. There are two main reasons: (1) In the steady state test, the degradation of tensile strength may have been further accelerated by the additional 30 min soaking time [27]; (2) In the transient test, the temperature at the surface part of CFRP tendons was higher than that in the internal part.

In the transient tests, the failure mode of CFRP tendons is shown in Figure 7b. For the specimens under loads of 50–70 kN, the time from the beginning of the test to the failure of CFRP tendons was shorter. The tendons were still characterized by brittle fracture at failure. This was mainly attributed to the fact that most of the undecomposed resin still had an effective bonding effect, while for the specimens under loads of 30–40 kN, the resin was obviously decomposed, and the duration time of the test was longer before the failure of CFRP tendons. 

## 4. Modeling of Strength Properties at Elevated Temperatures

### 4.1. Description of Models

In the present section, the experimental results of CFRP tendons at elevated temperatures are used to evaluate the applicability and accuracy of the models proposed in different literatures. In order to fully reveal the influence of thermo–physical responses on thermo–mechanical properties, a new model is proposed with the form of exponential function.

According to the characteristics of FRP composites at elevated temperatures, the temperature (*T_ref_*) at which the tensile strength and Young’s modulus vanish and the room temperature (*T*_0_) are applied in the expression by Gu [28]. The equation is provided as follows:

(2) $$P(T)={\left(1-\frac{T-T_{0}}{T_{ref}-T_{0}}\right)}^{m}\cdot P_{0}$$

where *P*(*T*) and *P*_0_ represents the mechanical property at *T* (°C) and room temperature, respectively; The value of the power law index *m* can be chosen between 0 and 1.

Subsequently, in order to fit the curve simply and accurately, a cubic polynomial function was proposed by Liu [29]. The glass temperature was successfully applied in the expression as follows:

(3) $$P(T)=[1-a_{1}(\frac{T-T_{0}}{T_{g}-T_{0}})-a_{2}{\left(\frac{T-T_{0}}{T_{g}-T_{0}}\right)}^{2}-a_{3}{\left(\frac{T-T_{0}}{T_{g}-T_{0}}\right)}^{3}]\cdot P_{0}$$

More recently, in the study of mechanical properties of GFRP composites at elevated temperatures, Correia [12] proposed an exponential function model which was derived from Gompertz statistical distribution theory.

(4) $$P(T)=(1-e^{Be^{C\times T}})\times (P_{0}-P_{r})+P_{r}$$

in which coefficients B and C are determined by experimental data; *P*_0_ and *P_r_* are the mechanical properties of the composites at ambient temperature and in the decomposed state, respectively.

As mentioned in the previous section, the critical temperature can be used as a criterion for evaluating the fire resistance of strengthened concrete members. On the basis of Gibson [14] model, Yu [6] further proposed the following hyperbolic tangent function model.

(5) $$P(T)=(\frac{P_{0}+P_{R}}{2}-\frac{P_{0}-P_{R}}{2}\tanh(k(T-T_{c}))$$

where *P_R_* is the relaxed (high temperature) value of the mechanical properties; *k* is a constant describing the extent of relaxation; *T_c_* is critical temperature of CFRP tendons.

In order to reveal the thermophysical properties of CFRP composites at elevated temperatures, a modified model is suggested by the authors, which is based on the exponential function model. Equation (6) contains the comprehensive effects of fiber content, resin softening, and decomposition on the mechanical properties of CFRP composites at elevated temperatures.

(6) $$\frac{P(T)}{P_{0}}=(1-V_{f})\exp\left[-k_{1}{\left(\frac{T-T_{0}}{T_{g}}\right)}^{3}\right]+V_{f}\exp\left[-k_{2}{\left(\frac{T-T_{0}}{T_{d}}\right)}^{3}\right]$$

where *k*_1_ and *k*_2_ are constants determined by fitting the experimental data; *V_f_* is the volume fractions of fibers.

### 4.2. Results and Discussion

Table 5 summarizes simulation results of strength degradation values of CFRP tendons with different models. For the empirical models, the parameters were determined by least squares regression analysis. In Gu and Asaro’s model, *T_ref_* (597 °C and 578 °C) was obtained by linear interpolation according to Table 3 and Table 4. In Equation (4), Correia defined *P_r_* as the mechanical property after glass transition (but before decomposition), corresponding to the strength in the leathery state. However, the experimental results in this paper show that it was more reasonable to refer to *P_r_* as the strength in the decomposed state. Thus, *P_r_* had the same meaning as *P_R_* in Yu’s model. As discussed above, the resin of CFRP tendons was completely decomposed in steady state and transient tests at 518 °C and 468 °C, respectively. Therefore, the strength at 518 °C and 468 °C was used as *P_r_* or *P_R_*.

In statistics, the coefficient of determination (R-Square) can represent the accuracy of a fitting model. The normal range of its value is between 0 and 1. The closer R-Square approaches 1, the better the model fits with the data. The comparison of different models shows that Liu model and the model proposed in this paper have better fitting accuracy. At the same time, the proposed model not only has fewer parameters, but also reflects the thermophysical properties of CFRP composites.

**Figure 9:**
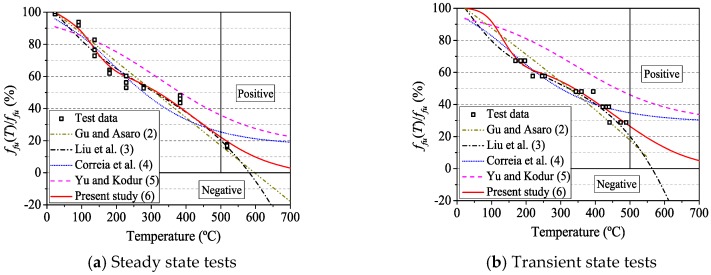
Nominal strength–temperature modeling curves.

The strength degradation curves derived from different empirical relations are plotted in Figure 9. All the models were in good agreement with the experimental data in the range of temperatures analyzed. It is a fact that the mechanical properties of CFRP composites after the decomposition of the resin approached zero. However, when the temperature exceeded 500 °C, the results fitted by the Gu model and the Liu model became negative, which did not satisfy with the physical truth. Again, it confirms the applicability and accuracy of the proposed model in this paper for calculating the mechanical properties of CFRP composites at elevated temperatures.

## 5. Conclusions

In this paper, detailed experimental studies are presented on the material properties of CFRP tendons at elevated temperatures. Based on the experimental data obtained, the fitting accuracy of different strength prediction methods is evaluated. The main conclusions can be drawn as follows:

(1) In the thermal expansion tests, experimental results reported herein confirmed that the longitudinal deformation of CFRP tendons is shrinkage at elevated temperatures. The coefficient of thermal expansion decreases with the increase of temperature. The CTE of CFRP tendons predicted by polynomial function is in good agreement with the experimental data before 300 °C.
(2) In the steady state tests, the tensile strength of CFRP tendons shows a characteristic of degradation with temperature. Especially, in the temperature range of glass transformation and decomposition of the resin, it decreases rapidly. When heated 324 °C, the retained strength of CFRP tendons is 50%. Hence the critical temperature of CFRP tendons in the steady state tests is 324 °C.
(3) The transient temperature test is more representative for actual fire conditions than the steady state test. The fire resistance time of CFRP tendons increases with the decrease of loading level. Corresponding to the steady state test results, the critical temperature of CFRP tendons in the transient state tests is 341 °C.
(4) Based on experimental data, all models suggested in different literatures can reasonably simulate the variation of tensile strength in the temperature range of the test. The new model proposed in the present study seems to be particularly well suited for describing the strength degradation of CFRP tendons as a function of temperature. It can be used in evaluating fire response of concrete members strengthened with CFRP tendons.

## Figures and Tables

**Table 1:** Material properties of carbon fiber reinforced polymer (CFRP) tendons used.

Specimen	Dimension		Tensile Properties		Physical Properties			
	Length L(mm)	Diameter D(mm)	Strength $f_{fu}$ (MPa)	Modulus $E_{f}$ (GPa)	Density $\rho_{f}$ (g/cm^3^)	Fiber Content $V_{f}$ (%)	*T_g_* (°C)	*T_d_* (°C)
CFRP	800	8	2070	156	1.6	65	126	405

**Table 2:** Number of specimens in this study.

Type of Tests	Number of Specimens
Thermal expansion test	3
Steady state test	24
Transient state test	15
Total	42

**Table 3:** Tensile strength of CFRP tendons in steady state tests.

Target Temp (°C)	Average Surface Temp (°C)	Strength (MPa)			Average Strength (MPa)	Standard Deviation	Coefficient of Variation	$f_{fu}(T)/f_{fu}$ %
		SS-1	SS-2	SS-3				
20	23	2048	2087	2075	2070	16.3	0.0079	100
100	91	1906	1943	1901	1917	18.7	0.0098	92.6
150	137	1584	1714	1508	1602	85.1	0.053	77.4
200	180	1325	1303	1281	1303	18.0	0.014	62.9
250	228	1248	1165	1097	1170	61.7	0.053	56.5
300	278	1105	1110	1091	1102	8.0	0.0073	53.2
400	383	996	901	953	950	38.8	0.041	45.9
500	518	331	365	350	349	13.9	0.040	16.9

**Table 4:** Failure temperatures of CFRP tendons in transient state tests.

Target Load (kN)	Stress (MPa)	Stress Ratio (%)	Temp (°C)			Average Temp (°C)
			TS-1	TS-2	TS-3	
30	597	28.8	441	489	473	468
40	796	38.5	432	421	439	431
50	995	48.1	360	344	394	366
60	1194	57.7	253	246	219	239
70	1393	67.3	169	196	184	183

**Table 5:** Simulation of CFRP material strength for different models.

Model	Parameters	Steady State		Transient State	
		Strength	R-Square	Strength	R-Square
Gu and Asaro [28]	*m*	1	0.941	0.885	0.782
Liu et al. [29]	*a*_1_	0.281	0.963	0.332	0.957
	*a*_2_	−0.056		−0.09	
	*a*_3_	0.007		0.012	
Correia et al. [12]	B	−3.529	0.936	−2.862	0.898
	C	−0.007		−0.007	
Yu and Kodur [6]	*k*	0.0035	0.796	0.0036	−0.548
Present study	*k*_1_	1.419	0.984	1.155	0.955
	*k*_2_	0.663		0.552	
